# Detection and Control of Foodborne Pathogens

**DOI:** 10.3390/foods12193521

**Published:** 2023-09-22

**Authors:** Antonello Paparella, Francesca Maggio

**Affiliations:** Department of Bioscience and Technology for Food, Agriculture and Environment, University of Teramo, Via Balzarini 1, 64100 Teramo, Italy; fmaggio@unite.it

## 1. Introduction

The globalization of food trade and the emergence of disease outbreaks involving several foodborne pathogens and foods has focused the attention of both the research community and consumers on food safety. Microbial contamination can involve different stages of food processing and distribution, with a potentially dramatic impact on human health and food business. Several methods, involving culture-dependent and -independent techniques, were developed to detect foodborne pathogens in the food supply chain. In this respect, gold-standard reference methods are currently available for most pathogens, but some of them are time-consuming and expensive. Moreover, routine controls carried out by manufacturers and food safety authorities are normally focused on bacteria and not on viruses and fungi, which can have a significant impact on food safety. In the European Union, the number of human cases caused by Noroviruses and other Caliciviruses increased sharply in 2021 [1]. Therefore, based on the evidence given by an increasing amount of research, control strategies show a clear trend towards molecular techniques, such as polymerase chain reaction (PCR), multiplex PCR, real-time PCR (qPCR), reverse transcriptase PCR (RT-PCR), DNA microarrays, nucleic acid sequence-based amplification (NASBA), isothermal DNA amplification techniques, and next-generation sequencing (NGS) [2]. Moreover, to expand the knowledge on the behaviour of pathogens in food environments, proteome profiling and biosensors shed light on how these microorganisms interact and prevail in food systems [3].

## 2. Microbial Ecology and Foodborne Pathogens 

The conditions imposed by the physical and chemical characteristics of food products, as well as by the hurdles adopted in food manufacturing, act as selective factors for the survival and the growth of pathogens [4]. However, hurdles that were believed to be decisive for food safety, e.g., low water activity (a_w_), are now known to allow the persistence of pathogens [5]. Dominant species can interact among themselves and with the food matrix, with varying effects on the microbial ecology of foods. In fact, the interactions between microorganisms can be synergistic or antagonistic, determining protection in stressful environments or competition for nutrients, respectively [4,6]. Interactions in microbial communities are coordinated by a cell–cell communication system, called quorum sensing (QS), which induces behavioural changes in relation to population density. The QS system can control both cooperative and competitive behaviours in microbial communities. The QS-regulated products, such as secreted proteases, can be used by any member of the community and control the social balance between microbial cooperators, cheaters, and policers [7,8]. On the other hand, the QS system manages the virulence products, such as the secreted or cell-targeted toxins, to promote competition with other strains or species of bacteria [8].

## 3. Control of Foodborne Pathogens by Emerging Technologies 

The control of food-borne pathogens in food systems has evolved over time to promote the safety of food products and extend their shelf-life. The main control methods aim to inhibit the growth of foodborne pathogens by chemical and physical approaches. However, many of these methods affect the characteristics of food products, show low effectiveness over time, have a significant environmental footprint, and are not appreciated by consumers. New biocontrol methods have been proposed to maintain food appearance and acceptability, to preserve nutrients and to extend shelf-life [9]. Among these, emerging technologies such as ozone, pulsed light, high-hydrostatic pressure and high-pressure homogenization showed significant antimicrobial effectiveness [9,10,11,12]. Moreover, plant-derived compounds, such as essential oils and hydrolates, inhibit the growth of several microbial species, acting on multiple cellular targets and limiting bacterial defence mechanisms and therefore the occurrence of antimicrobial resistance [13].

## 4. Epidemiology of Emerging Foodborne Diseases

Surveillance is a long- or short-term control and management measure, aimed to monitor foodborne pathogens and their socio-economic impact, which includes the systematic collection of relevant incident data, disease frequency and additional factors regarding the incidence and the spread of outbreaks [14]. Tracking global threats is one of the main goals of surveillance programs at the international and local levels; this activity is fundamental to improving disease control measures and to establishing therapeutic guidelines, based on data collected by voluntary or mandatory participation [15]. It is estimated that 600 million humans become ill annually after eating contaminated food, and 420,000 die every year, causing economic losses of over USD 100 billion [16]. *Salmonella enterica*, *Campylobacter jejuni*, enterohaemorrhagic *Escherichia coli* and *Listeria monocytogenes* are the most common foodborne pathogens in human cases [16,17]. However, there is increasing evidence that other emerging or unknown pathogens can play an important role in foodborne diseases, such as *Aliarcobacter* spp., *Cronobacter* spp., *Vibrio* spp., *Clostridioides difficile*, *Mycobacterium paratuberculosis*, *Streptococcus suis*, *Helicobacter pylori*, and *Yersinia enterocolitica* [17]. Resistance to antimicrobial treatments and a probable underestimation of the real incidence of the emerging foodborne diseases increase the risk scale and the need to control old and new foodborne pathogens.

This Special Issue aims to publish quality articles on the detection and control of foodborne pathogens, also considering the impact of innovative technologies and the emergence of new foodborne pathogens.
